# Genome-Wide Identification and Characterization of MicroRNAs and Target Genes in *Lonicera japonica*

**DOI:** 10.1371/journal.pone.0164140

**Published:** 2016-10-06

**Authors:** Heng Xia, Libin Zhang, Gang Wu, Chunhua Fu, Yan Long, Jun Xiang, Jianping Gan, Yanhong Zhou, Longjiang Yu, Maoteng Li

**Affiliations:** 1 College of Life Science and Technology, Huazhong University of Science and Technology, Wuhan, Hubei 430074, China; 2 Institute of Biotechnology, Chinese Academy of Agricultural Sciences, Beijing, 100081, China; 3 Hubei Key Laboratory of Economic Forest Germplasm Improvement and Resources Comprehensive Utilization, Huanggang Normal University, Huanggang 438000, China; Kunming University of Science and Technology, CHINA

## Abstract

MiRNAs function in post-transcriptional regulation of gene expression and play very important roles in plant development. *Lonicera japonica* is one of the important medicinal plants in China. However, few studies on the discovery of conserved and novel miRNAs from *L*. *japonica* were reported. In this study, we employed deep sequencing technology to identify miRNAs in leaf and flower tissues of *L*. *japonica*. A total of 22.97 million clean reads from flower and leaf tissues were obtained, which generated 146 conserved miRNAs distributed in 20 families and 110 novel miRNAs. Accordingly, 72 differentially expressed miRNAs (P≤0.001) between leaves and flowers and their potential target genes were identified and validated. The qRT-PCR validation showed that majority of the differentially expressed miRNAs showed significant tissue-specific expression in *L*. *japonica*. Furthermore, the miRNA-mRNA and mRNA-mRNA regulatory networks were constructed using Cytoscape software. Taken together, this study identified a large number of miRNAs and target genes in *L*. *japonica*, which not only provides the first global miRNA expression profiles, but also sheds light on functional genomics research on *L*. *japonica* in the future.

## Introduction

MiRNAs are small non-coding RNA molecules, which regulate the gene expression at the post-transcriptional level by degrading or inhibiting the translation of targeted gene mRNAs [1, 2]. MiRNAs usually regulate gene expression by binding to the RNA-induced silencing complex (RISC) [1]. In plants, miRNAs regulate their target mRNAs by nearly perfect complementary base pairing, which leads to the degradation of target genes [3]. Many studies have reported that miRNAs play important roles in various biological processes, such as root development, leaf development, signal transduction and cell proliferation [4–6].

A majority of miRNAs are evolutionary conserved, which can be identified by sequence homology analyses [7]. Nevertheless, a proportion of miRNAs are species-specific and usually express at lower levels in comparison with other conserved miRNAs [8, 9]. It is difficult to identify the species-specific miRNAs by traditional experimental approaches due to their low expression levels. However, in the recent years, deep sequencing technology has been developed as an effective analysis tool to identify species-specific or low-abundance miRNAs in diverse plant species, such as *Arabidopsis* [9], *Zea mays* [10], *rice* [11], *Brassica napus* [12], *Chinese cabbage* [13], *potato* [14, 15] and *tomato* [16]. More importantly, this powerful strategy does not rely on the genome information of the specific species. Therefore, deep sequencing has been widely applied to the discovery of lowly expressed or species-specific miRNAs in non-model species without genome information.

*L*. *japonica* is a perennial, evergreen, twining vine. It is widely cultivated in eastern Asia including China, Japan and Korea as an effective groundcover because of its pleasant, sweet smelling flowers. In China, the *L*. *japonica* (also called as Jinyinhua) has been an important medicinal plant for thousands of years [17]. For example, *L*. *japonica* is often used to treat some human diseases, such as severe respiratory syndromes, H1N1 flu and hand-foot-and-mouth disease [18]. Meanwhile, *L*. *japonica* is also used as food and a worldwide healthy beverage, which recently leads to the rapid increase of the FLJ commercial value in the herbal medicine trading markets. Although next-generation sequencing technology has been applied for the transcriptome studies in *L*. *japonica*, to our knowledge, there is no report of identified microRNAs of *L*. *japonica* yet, which hindered the functional genomics and anti-disease researches of *L*. *japonica*. Therefore, there is an urgent need to identify the miRNAs of *L*. *japonica* in the genome-wide level.

In this study, we constructed the small RNA cDNA libraries from *L*. *japonica* leaf and flower and performed small RNA sequencing using Illumina Hi-seq 2000 platform. By taking advantage of bioinformatics approach, the conserved and novel *L*. *japonica* miRNAs were identified, respectively. Additionally, the potential target genes of the conserved and novel miRNAs were predicted. Annotation and network analysis of miRNA and their putative target genes were also performed to get a better understanding of their functions in various biological processes in *L*. *japonica*.

## Materials and Methods

### Plant material and RNA isolation

*L*. *japonica* plants were grown in experimental field of Huazhong University of Science and Technology, Wuhan, Hubei Province, China (Huazhong University of Science and Technology has issued the permission to collect *L*. *japonica* samples). The flowers and leaves of *L*. *japonica* were collected and immediately frozen in liquid nitrogen. RNAprep Pure Plant Kit (TIANGEN, Cat.#DP441) was used to isolate total RNAs of the flowers and leaves according to the manufacturer’s instructions. The isolated total RNAs were quantified by NanoDrop spectrophotometer (Thermo Fisher Scientific, Inc). The obtained RNA samples were stored in -80°C freezer for further study.

### sRNA library construction and deep sequencing

Small RNA library construction was performed as previously described [19]. Total RNAs of *L*. *japonica* were first size-fractioned on 15% PAGE gel and 18–30 nt fraction was collected. T4 RNA ligase was used to ligate the 5’ and 3’ RNA adapters to the RNA pool. The purified small RNAs with 5’ and 3’ adaptors were reverse-transcribed and amplified with PCR. The obtained cDNA samples were sequenced using Illumina Hi-seq2000. The clean reads were obtained by removing adaptor sequences, reads with low-quality (quality score < 20 and/or two more Ns), and/or short length (<14nt). The sequenced sRNA sequences were filtered by removing rRNA, tRNA, snRNA, snoRNA and materials containing the poly-A tail.

### Identification of conserved and novel miRNAs

To obtain the clean reads, we eliminated the low quality and contaminated reads according to the following rules: (I) the empty and low quality reads were first removed; (II) The adaptor sequences were trimmed; (III) The reads with 5’ primer contaminants and poly A were removed; (IV) The reads without 3’ primer were removed; (V) the reads shorter than 18 nt and longer than 30 nt were removed. The generated clean reads were mapped to *L*. *japonica* EST and GSS databases using SOAP [20]. The perfectly mapped sRNAs were used for the further studies, while the clean reads mapped to noncoding RNAs, such as rRNAs, tRNAs, snRNAs and snoRNAs in the GenBank [21] and Rfam [22] databases were removed. Then the remaining sRNAs were aligned with the mature miRNA or miRNA precursor in the miRBase [23] with a maximum of two mismatches. Only perfectly matched sRNAs were retained and considered to be conserved miRNAs. Also, the secondary structure, the Dicer cleavage site and the minimum negative folding free energy of the unannotated sRNAs mapped to *L japonica* EST and GSS databases were analyzed by the miRDeep2 software [24]. Only the sRNAs that fit all the published criteria [25] were considered to be novel miRNAs. For the novel miRNAs, the names from lcja-novel-miRx1 to lcja-novel-miRx124 were given [26].

### Differential expression analysis of miRNAs

To analyze the differentially expressed miRNAs, we compared the conserved and novel miRNA expression levels between flowers and leaves by calculating TPM (Transcripts per million). The differentially expressed miRNAs between flowers and leaves were determined with log FC (log-fold expression change) greater than 1 or less than -1 (FDR<0.001, p-value < 0.005).

### MiRNA expression analysis by qRT-PCR

To validate the small RNAs sequencing data and differentially expression of miRNAs between the leaf and flower tissues in *L*. *japonica*, randomly selected miRNAswere selected for qRT-PCR analysis. For cDNA synthesis, 3ug of total RNA from the leaf and flower were reverse respectively transcribed using kit (#AMRT-0020, GeneCopoeia) in S1000^™^-PCR instrument (#S1000,BIO-RAD). The qRT-PCR experiments of conserved miRNAs and predicted new miRNAs in leaves and flowers were performed in StepOne Plus PCR instrument (ABI) using miRNA qRT-PCR kit (#AMPR-0200, GeneCopoeia). U6 and 5s RNA was used as the internal control in these experiments. The relative expression fold changes of miRNAs were calculated using the comparative CT method. All reactions were performed in triplicate with three independent experiments. The primer sequences were listed in S1 Table.

### Prediction and annotation of target genes of miRNAs

The Mireap software (https://sourceforge.net/projects/mireap/) was employed to predict the miRNA targets basing on the published rules [27, 28]. The Gene Ontology tool (http://www.Geneontology.org/) was used for the annotation of the miRNA targets.

### Validation of the miRNAs cleavage site by 5′RLM-RACE (5′RNA ligase-mediated rapid amplification of cDNA Ends)

5′ RLM-RACE was used to identify the cleavage sites on the predicted miRNA targets. 5′ RLM-RACE was performed using First choice RLM-RACE kit (Ambion, AM1700), according to the instructions of the manufacturer. Gene-specific reverse primers and gene-specific reverse nested primers (S1 Table) were designed from the predicted targets and used in combination with the 5′adapter primers to amplify the cleaved transcripts. The target fragments were cloned into pTA2 vectors (TOYOBO, TAK101) and sequenced using the universal T7 primers.

## Results

### Sequence analysis of sRNA libraries

**Fig 1:**
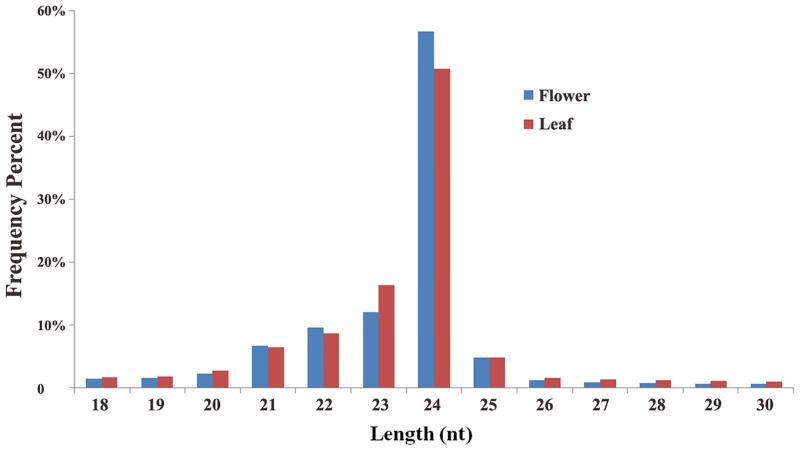
Length distribution and abundance of small RNAs from *L*. *japonic*a flower and leaf.

In order to identify conserved and novel miRNAs, total RNA was isolated from flowers and leaves in *L*. *japonic*a. The cDNA libraries of flowers and leaves were then constructed independently. Furthermore, small RNA sequencing generated 12.95 million clean reads and 10.02 million clean reads from flower and leaf libraries (Table 1), respectively. The obtained clean reads were deposited in SRA (NCBI Sequence Read Archive) database under accession number of SRP067769 and SRP067770. The length distribution of the small RNAs was shown in Fig 1. Among them, the ratios of 20–24 nt small RNAs in the flower and leaf tissues of *L*. *japonic*a were 87.45% and 84.95%, respectively. Moreover, the ratios of 24 nt small RNAs in flowers and leaves were 56.62% and 50.72%, respectively. This result is consistent with previous reports in some other plant species including *Arabidopsis* [9], *rice* [11] and *peanut* [29]. The clean sRNA reads were then mapped to the *L*. *japonica* transcriptome sequence (SRP052594). The total mapped reads *vs* uniquely-mapped were 5,328,437/505,860 in the flower library and 4,284,236/687,957 in the leaf library (Table 1). Furthermore, we removed the rRNAs, tRNAs, snRNAs and snoRNAs in the two sRNA libraries. As shown in Table 1, rRNAs and tRNAs were the most abundant small RNAs in the two sRNA libraries. In the meanwhile, to verify and remove the sRNAs distributed in the CDS (coding sequence) regions, we compared the clean sRNA reads with the CDS sequences of *Arabidopsis thaliana*. The result showed that the number of total and unique reads that mapped to the CDS region were 12,019/2,230 in the flower library and 7,668/2,093 in the leaf library. In order to investigate which sRNAs are tissue-specific in *L*. *japonic*a, we performed a comparative analysis of the clean sRNA reads between the flower and leaf tissues. As a result, the majority of the clean reads (10,779,174 reads) were detected both in flower and leaf tissues, while 6,717,226 and 5,475,753 reads were specifically detected in flower and leaf, respectively. In addition, we also analyzed the unique sRNA reads between flower and leaf tissues. The result showed that 267,935 unique reads were detected in both tissues. Whereas, 3,372,126 unique reads were specifically found in flower and 3,104,768 unique reads were specifically found in leaf.

**Table 1:** Statistical analysis of small RNA sequencings for flower and leaf in *L*. *japonica*.

	Flower	Leaf
**Clean reads**	12,949,668	10,022,485
**Mapped to *L*. *japonica* transcriptome**	5,328,437	4,284,236
**Uniquely-mapped reads**	505,860	687,957
**rRNA**	416,641	154,014
**tRNA**	327,712	264,622
**SnoRNA**	5,735	7,184
**snRNA**	3,764	6,018

### Identification of conserved miRNA in *L*. *japonica*

In order to identify the conserved miRNA in *L*. *japonica*, the sRNA sequences generated from flower and leaf were compared with the known plant miRNAs in the miRBase database. A total of 146 known miRNAs that distributed in 20 families were identified in the flower and leaf libraries (S2 Table). Among these miRNA families, 16 families were represented in both tissues. Whereas, miR393 and miR160 families were only observed in leaf, while miRNA172 and miRNA 171 families were only identified in flower.

The read numbers for conserved miRNA families showed dramatic variations (S2 Table), ranging from 20 to 109,007,831 in the flower tissue and 428 to 103,630,819 in the leaf tissue. In our study, for 10 highly conserved miRNA families (miR156, miR159, miR162, miR164, miR166, miR167, miR168, miR319, miR396, miR403), more than ten thousand reads were detected in flowers and leaves. Among them, miR166 family has the most sequencing reads in flowers and leaves. Meanwhile, for 8 moderately conserved miRNA families (miR160, miR171, miR172, miR393, miR394, miR398, miR408, miR6149), more than one thousand reads were found at least in one tissue. Interestingly, miR393 was expressed at a very low abundance with 20 reads in the flower tissue, which could be considered as low-abundance conserved miRNAs. It has been reported that low-abundance conserved miRNAs play crucial roles in plant developmental and cellar processes, such as flower development and abiotic stress responses [30].

### Identification of novel miRNA in *L*. *japonica*

The characteristic hairpin structure of miRNA precursor is an essential feature for predicting novel miRNAs [31]. As the full genome information of *L*. *japonica* is unavailable, the unique sRNA sequences of flowers and leaves were mapped to *L*. *japonica* EST and GSS sequences. In order to identify novel miRNAs in *L*. *japonica*, the potential miRNA precursor sequences were folded by miRDeep2 software. The Dicer cleavage site and the minimum negative folding free energy were also employed to evaluate the candidate miRNAs. As a result, we detected a total of 110 novel miRNAs expressed in the flowers and leaves (S3 Table). Meanwhile, 11 novel miRNAs were observed in both tissues, whereas 58 and 41 novel miRNAs were specifically expressed in flower and leaf tissue, respectively (S3 Table). The length of miRNAs in flowers ranged from 18 to 24 nt, while the length of miRNAs in leaves ranged from 18 to 25 nt. Moreover, the miRNAs with 24 nt were most abundant in both samples, followed by the miRNAs with 21 nt. The length of the novel miRNA precursors ranged from 44 to 228 nt in the flower sample and 38 to 224 nt in the leaf sample, with an average of 94 nt and 85 nt, respectively. The negative folding free energies of the novel miRNA precursors ranged from -84.8 to -11 kcal / mol. The average negative folding free energy is -36.38 kcal/mol, which is different from that in tRNA and rRNA [32]. In comparison with the conserved miRNAs, the novel miRNAs in *L*. *japonica* showed much lower expression levels (S3 Table). The majority (89.1%) of these novel miRNAs were sequenced less than 100 reads.

### qRT-PCR validation and differential miRNA expression analysis between flower and leaf

To identify the differentially expressed miRNAs between flower and leaf in *L*. *japonica*, we analyzed the expression pattern of miRNAs between the two tissues by calculating TPM. The miRNA expression level of flower was defined a control to obtain the up- or down-regulated miRNAs in leaf. As a result, a total of 72 differentially expressed miRNAs (DE-miRNAs) were identified between leaf and flower, including 33 miRNAs (FDR<0.001) with up-regulated expression pattern and 39 miRNAs (FDR<0.001) with down-regulated expression pattern (S4 Table). In addition, there were 46 DE-miRNAs observed only in leaf, while 33 DE-miRNAs were only expressed in flower.

**Fig 2:**
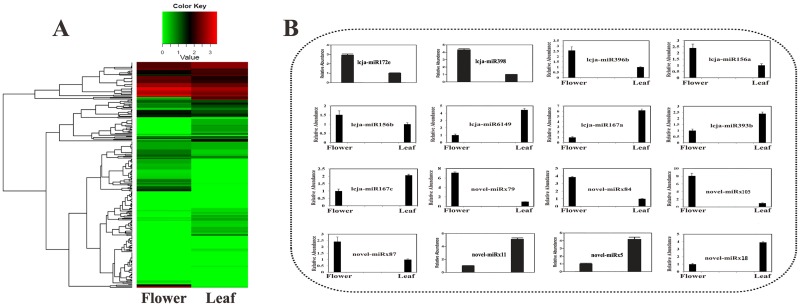
(A). Heatmap analysis of miRNA expression between flower and leaf. (B). Quantitative RT-PCR Validation of differentially expressed miRNAsbetween flower and leaf.

In order to verify the data obtained from small RNA sequencing, we further selected some miRNAs for qRT-PCR analysis. The qRT-PCR experiment results were consistent with the small RNA sequencing data (S4 Table). The majority of analyzed miRNAs showed significant tissue-specific expression in *L*. *japonica* (Fig 2A and 2B). For example, lcja-miR6149, lcja-miR167a, lcja-miR393b, novel-miRx11, novel-miRx5 and novel-miRx18 exhibited lower expression in flower than in leaf, while lcja-miR396b, lcja-miR156a, lcja-miR172e, lcja-miR398, novel-miRx79, novel-miRx84, novel-miRx87 and novel-miRx105 displayed higher expression in flower than in leaf, which indicates the developmental tissue-specific regulation of miRNA expression in *L*. *japonica*.

### Prediction, validation and annotation of miRNA target genes in *L*. *japonica*

**Fig 3:**
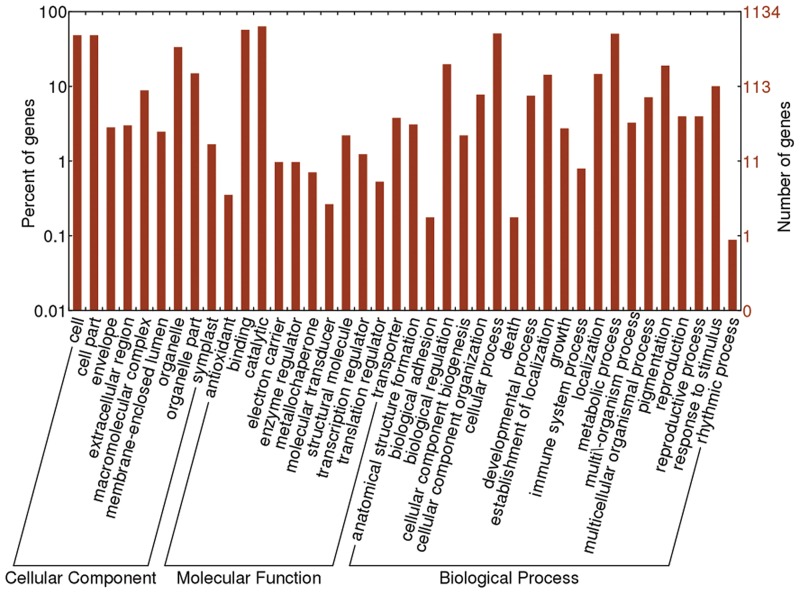
The GO annotation results of potential miRNA target genes in *L*. *japonica*.

The potential target genes of miRNAs in *L*. *japonica* were predicted using Mireap according to the published criteria [27, 28]. The *L*. *japonica* transcriptome data (SRP052594) was used as reference set. As a result, 2,035 target genes were predicted for 126 miRNAs (S5 Table). The number of target genes per miRNA varied from 1 to 423, with an average number of 16.2. Meanwhile, two predicted miRNA targets were selected for validation using 5′ RLM-RACE. As shown in S1 Fig, 2 miRNA cleavage sites were detected on their mRNA targets by 5′ RLM-RACE. To analyze the function of these potential target genes, all the targets genes were assigned with GO terms, which were classified into 40 functional groups distributed under three main categories including cellular component (1,858 sequences), biological process (2,518 sequences) and molecular function (1,491 sequences) (Fig 3). Within the cellular component category, “cell” (548 sequences), “cell part” (548 sequences), “organelle” (380 sequences) and “organelle part” (169 sequences) were enriched. Among the biological process category, “cellular process” (578 sequences), “metabolic process” (573 sequences), “biological regulation” (224 sequences) and “pigmentation” (215 sequences) were major GO terms. In the molecular function category, “catalytic activity” (719 sequences) and “binding” (647 sequences) were the most abundant. Meanwhile, to investigate the biological functions and metabolic pathways of the target genes, the potential target genes were further annotated with KEGG database. A total of 81 KEGG pathways were obtained as shown in (S6 Table). Taken together, the results showed that these potential target genes were involved in various biological processes, which indicated that miRNAs play important regulatory roles in the growth and development processes of *L*. *japonica*.

### Integrative analysis of miRNA and mRNA networks in *L*. *japonica*

**Fig 4:**
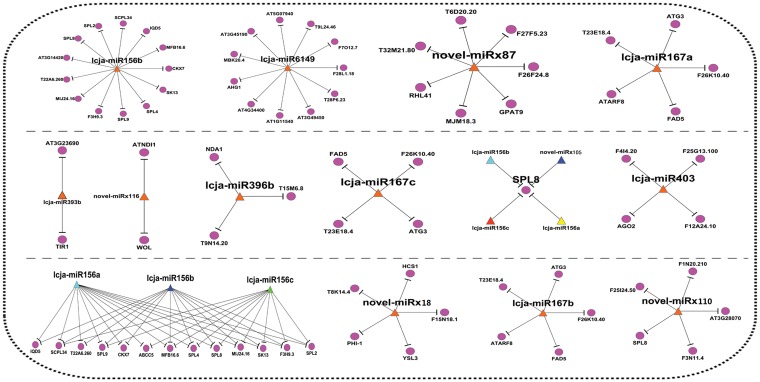
miRNA-mRNA regulatory network analysis in *L*. *japonica*.

MiRNAs play important roles in the regulation process of plant growth and development. Here, we identified a total of 72 differentially expressed miRNAs (DE-miRNAs) between leaf and flower, including 51 novel miRNAs. Furthermore, we explored the miRNA-mRNA regulatory network through the integration analysis of miRNA and mRNA in *L*. *japonica*. Fig 4 showed some important miRNA-mRNA regulatory networks in *L*. *japonica*. For example, lcja-miR156b and lcja-miR6149 might be two important regulators, each of which targets more than 10 genes in the miRNA-mRNA network (Fig 4). For instance, SPL family proteins, the putative mRNA targets of lcja-miR156b, are important transcription factors and play regulatory roles in multiple biological processes of plants[33, 34]. Also, we observed RHL41 is a putative mRNA target of novel-miRx87, which is a transcription factor involved in the increased tolerance to high light and cold acclimation [35]. Our result (Fig 2B) showed that novel-miRx87 was down-regulated in leaf tissue of *L*. *japonica*, indicating that leaf might have more resistance to high light and cold acclimation than flower in *L*. *japonica*.

## Discussion

MiRNAs are important small non-coding RNA moleculesin regulating plant physiological process [1]. In recent years, next-generation sequencing technology has provided a popular method to identify conserved and novel miRNAs. However, few studies of miRNAs discovery have been reported in *L*. *japonica*, which is one of the most important medicinal plants widely cultivated in eastern Asia. In our study, two small RNA libraries from flowers and leaves in *L*. *japonica* were sequenced by next-generation sequencing technology. Approximately 12.95 million clean reads from the flower library and 10.02 million clean reads from the leaf library were obtained, respectively, in which 20 conserved miRNA families and 110 novel miRNAs were identified. In our study, the highly conserved miRNA families, such as miR156, miR159, miR162, miR164, miR166, miR167, miR168, miR319, miR396 and miR403 family, had at least ten thousand reads in the flower and leaf tissues, which was consistent with the report that plant evolutionary conservation had the correlation with expression abundance (32). These highly conserved miRNAs play important roles in the processes of plant growth and development. For instance, lateral root development and adventitious rooting were affected by miR164 and miR167 in *A*. *thaliana* [36], while the development of floral organs was regulated by miR159, miR166 and miR167 [37]. Moreover, we identified 8 moderately conserved miRNA families including miR160, miR171, miR172, miR393, miR394, miR398, miR408 and miR6149. These miRNA families were sequenced more than one thousand reads at least in one tissue, which suggested that they might play important roles in plant gene regulation [4–6].

In this study, we successfully identified 110 novel miRNAs by mapping and hairpin structure prediction. Novel miRNAs usually displayed relatively lower expression level and were difficult to be identified by traditional methods according to previous study [3]. Therefore, the prediction of miRNA targets is a crucial method to reveal the regulatory roles of miRNAs. Here, a total of 2,035 target genes were predicted by the high homology analysis between miRNAs and target sequences. The identified target genes were mainly involved in “cellular process”, “metabolic process” “biological regulation” and “pigmentation”.

**Fig 5:**
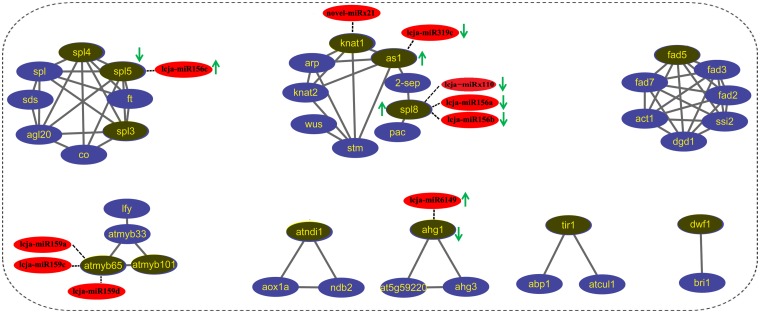
Construction of the interaction networks of mRNA targets by Cytoscape software. Blue ellipses represent the genes involved in the networks; Gray ellipses represent the genes involved in the networks; The arrows (up) indicate the upregulated miRNAs or targets *L*. *japonica*, while the arrows (down) indicates the downregulated miRNAs or targets in *L*. *japonica*.

Meanwhile, we found that some miRNA target genes are plant transcription factors such as WRKY and GRAS family proteins. For example, The WRKY proteins were reported to play significant roles in responses to biotic and abiotic stresses [38], while GRAS family proteins are a class of important transcription factors and play crucial roles in plant growth and development [39]. Furthermore, the miRNA-mRNA regulatory network was constructed through the integration analysis of miRNA and mRNA in *L*. *japonica* (Fig 4). We found lcja-miR156b and lcja-miR6149 might be two potential regulators, each of which targets more than 10 genes in the miRNA-mRNA network. Also, the potential interactions among the mRNA targets of these DE-miRNAs were established by Cytoscape software, in which we found some important interactions. As shown in Fig 5, we observed that 2-sep, knat1, arp, knat2 and stm directly interacts with as1 in the networks. Among them, 2-sep might act as one chloroplastic-like protein in Zea [40] and one potential light stress-regulated chlorophyll-binding protein in *Arabidopsis thaliana* [41], while as1 is usually involved in the process of leaf morphogenesis. Our q-PCR result (S2 Fig) showed that as1 was upregulated in leaf in comparison with flower and directly interacted with 2-sep, which suggested the important roles of the interaction between as1 and 2-sep in the process of leaf development. In addition, we observed that ft, co, sds, and agl20 interact with SPL family proteins (Fig 5). Moreover, SPL5 displayed a significant negative correlation with lcja-miR156c (S2 Fig). As SPL family proteins are plant-specific transcription factors and function in the process of flower and fruit development, plant architecture and so on [33, 34]. Therefore, we assume that the over-expression of the SPL5 in flower is associated with the regulatory roles in the process of flower development. Taken together, these results indicated that miRNAs may play crucial regulatory roles in tissue growth and development by various complex networks. Nevertheless, future studies are still necessary to explore the biological functions of miRNAs by regulation network analysis in *L*. *japonica*.

## Conclusions

In the present study, we identified 146 conserved miRNAs and 110 novel miRNAs using deep sequencing technology. This is the first report on such an analysis in *L*. *japonica*. Further analysis showed that majority of the differentially expressed miRNAs displayed significant tissue-specific expression in *L*. *japonica*. Additionally, the miRNA-mRNA and mRNA-mRNA regulatory networks were established by Cytoscape, which will help to explore the biological functions of miRNAs by regulation network analysis in *L*. *japonica*. Taken together, this study not only provides the first global miRNA expression profiles, but also lays the foundation for further research into the functional genomics of *L*. *japonica* in the future.

## Supporting Information

S1 Fig Quantitative RT-PCR Validation of differentially expressed miRNAs and mRNAs between flower and leaf. (TIF)

S2 Fig 5’ RLM-RACE analysis of the miRNA cleavage sites on their target mRNAs and Quantitative RT-PCR assay (A. lcja-miR398 and its target LHCA2; B. lcja-novel-miRx98 and its target GLDP2). (TIF)

S1 Table qRT-PCR primer sequences in *L*. *japonica*. (DOCX)

S2 Table Identification of conserved miRNA families in *L*. *japonica*. (XLS)

S3 Table Identification of novel miRNAs in *L*. *japonica*. (XLS)

S4 Table Differential miRNA expression analysis in *L*. *japonica*. (XLSX)

S5 Table Target of miRNAs analysis in *L*. *japonica*. (XLS)

S6 Table kegg annotation of target genes in *L*. *japonica*. (XLS)
