# Supplementary figures and images for: Genome-Wide Identification and Characterization of MicroRNAs and Target Genes in Lonicera japonica
Source: PLoS One. 2016 Oct 6;11(10):e0164140. doi: 10.1371/journal.pone.0164140 (PMC5053492; doi:10.1371/journal.pone.0164140)

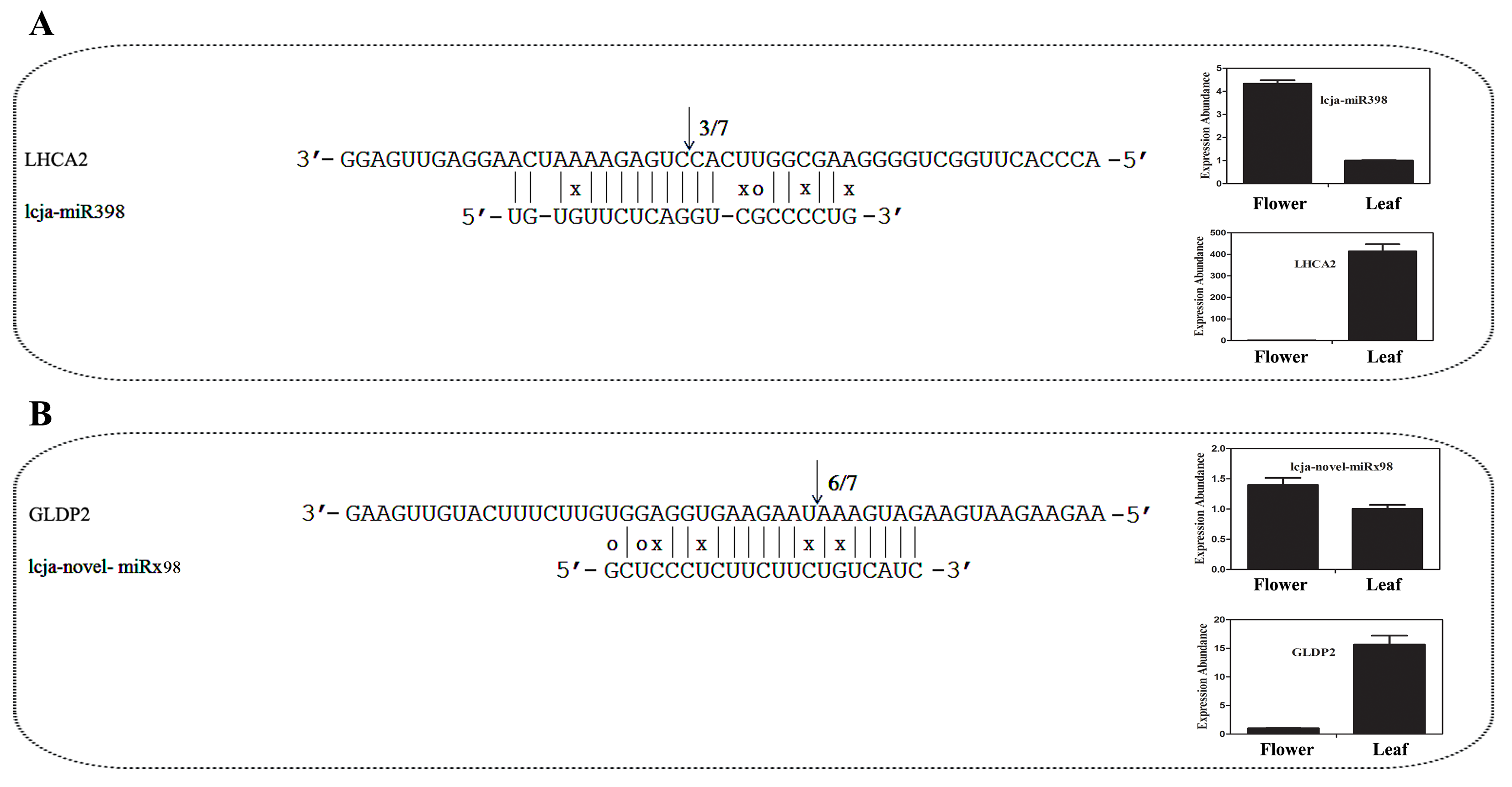

Supplement: S1 Fig — (TIF) [file pone.0164140.s001.tif]

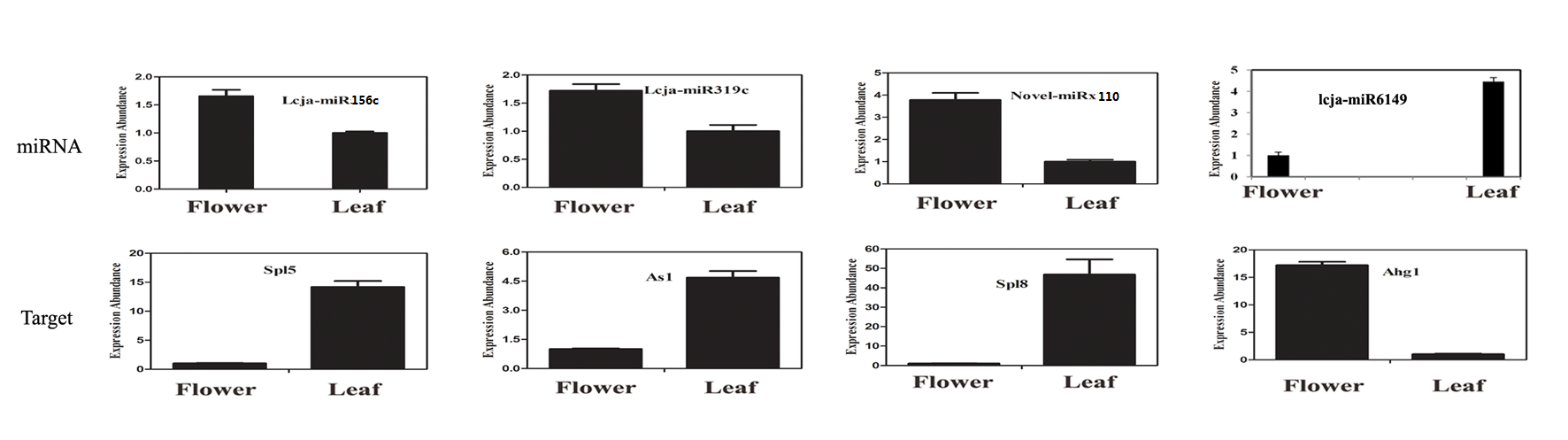

Supplement: S2 Fig — (TIF) [file pone.0164140.s002.tif]
