# Supplementary material for: Genome-Wide Identification and Characterization of MicroRNAs and Target Genes in Lonicera japonica
Source: PLoS One. 2016 Oct 6;11(10):e0164140. doi: 10.1371/journal.pone.0164140 (PMC5053492; doi:10.1371/journal.pone.0164140)
Supplement: S1 Table — (DOCX) [file pone.0164140.s003.docx]

**TABLE S1 Primers of miRNAs and target genes used for qRT-PCR validation in *L. japonic*a.**

| **miRNA name** | **Sense primer(5’→3’)** | **Anti-sense primer(5’→3’)** |
| --- | --- | --- |
| lcja-miR172e | GAATCTTGATGATGCTGCA | - |
| lcja-miR398 | TGTGTTCTCAGGTCGCCCCTG | - |
| lcja-miR396b | TTCCACAGCTTTCTTGAACTG | - |
| lcja-miR156a | TTGACAGAAGAGAGTGAGCAC | - |
| lcja-miR156b | TGACAGAAGAGAGTGAGCAC | - |
| lcja-miR6149 | TTGATACGCACCTGAATCGGC | - |
| lcja-miR393b | TCCAAAGGGATCGCATTGATC | - |
| lcja-miR167c | TGAAGCTGCCAGCATGATCTGA | - |
| lcja-miR171p | TTGAGCCGTGCCAATATCAC | - |
| lcja-miR156c | TTGACAGAAGATAGAGAGCAC | - |
| lcja-miR319c | CTTGGACTGAAGGGAGCTCC | - |
| novel-miRx79 | AATTCCACTCGGTTCTTTAGGATC | - |
| novel-miRx84 | GTGGACTCTTAATTTGGA | - |
| novel-miRx105 | CCAGATCTAACTCTTCCAGCTGA | - |
| novel-miRx87 | GCCCGTCTAGCTCAGTTGG | - |
| novel-miRx11 | TTAAGGGAGAGACTTAGACCT | - |
| novel-miRx5 | GTGTATTTAAGTCTCTGATGATT | - |
| novel-miRx73 | GTTTCTGATCTGGGTTTTA | - |
| novel-miRx18 | CAACATTCTTATGCACCCA | - |
| novel-miRx98 | GCTCCCTCTTCTTCTGTCATC | - |
| novel-miRx94 | CTGAGAGCTCTTTCTTGA | - |
| novel-miRx110 | AACGGATATGGTAAGAAAAGG | - |
| 5S | GGCTCGGCAACGGATATCTCG | - |
| **Gene name** |  |  |
| *SPL5* | AATCTACCCTTCTTAATTCACC | CTTCTGCCCAGCCCTATT |
| *AS1* | TTCAGTCACCCTTAGCCT | CCTTCTCCGATTCCAGTT |
| *SPL8* | ATCCTACCCACCTCCTCC | TGGAACTCGCAGACCTTAT |
| *Ahg1* | CAAGTGACGGTCTATGGG | AATCGACGACTATTACGCTA |
| AT1G18270 | TCAAATCCCAATTCAAGAAC | CCCGAAAGTAGACATCCAG |
| *LHCA2* | TTGATTGGTTGGGCTGAG | ATGGCGAGTCTTCCGTTT |
| comp134395_c0_seq6 | ATGGCTCAGATTGAACGC | AAGTTGTGGCATGACTGG |
| F11F8_15 | TCAATGCGGTTCAGGTCA | GATTAGGTGCTGGTAGGC |
| comp3060670_c0_seq1 | TGCATAAGAATGTTGGCTCA | AGCCCACTCTTGGTTATTTGC |
| GLDP2 | TGAAATAAGAAGTGGAGGTG | CAGACAAATGGAGCAAAC |
| comp55489_c0_seq1 | CGCATGGATACAAGTTAGG | GGGTATTAGCAGCCGTTT |
| comp55451_c0_seq1 | ATCCATCCATTTAGCAGCAC | TCAGTTCGCCAGGTTGTC |
| U6 | CGGGGACATCCGATAAAATTGG | TCTCGATTTATGCGTGTCATCCT |

**Primers of target genes used for 5’-RACE in *L. japonic*a.**

| **Gene name** | **primer(5’→3’)** |  |
| --- | --- | --- |
| 5'RACE Outer Primer-F： GCTGATGGCGATGAATGAACACTG | | |
| 5' RACE Inner Primer-F： GAACACTGCGTTTGCTGGCTTTG | | |
| comp55489_c0_seq1-R(out): TTTCAGATGAGTAATGTTGTTCT | | |
| comp55489_c0_seq1-R(in): GTCCGCCACTGGAAACAC | | |
| comp55451_c0_seq1-R(out): GCCACTTTGATCCCATTT | | |
| comp55451_c0_seq1-R(in): AGTTCGCCAGGTTGTCTC | | |
| LHCA2-R(out): AAGTTGTGGCATGACTGG | | |
| LHCA2-R(in): GGTTGTCAATGGGTCCTG | | |
| F11F8_15-R(out): AGCTTGGGACAGGTGGAT | | |
| F11F8_15-R(in): AGGAAGGTGAAGGAGGGA | | |
| GLDP2-R(out): AAGGCAAGCTCATTAACAACAA | | |
| GLDP2-R(in): TCCACCCTCGCTACTCAT | | |
| comp3060670_c0_seq1-R(out): GCTTGGGACAGGTGGATTG | | |
| comp3060670_c0_seq1-R(in): TTGAATCACTGAGTAGCG | | |
| comp134395_c0_seq6-R(out): AGTTGTGGCATGACTGGAT | | |
| comp134395_c0_seq6-R(in): CGTTACCTTGGTAGGCTTT | | |
| AT1G18270-R(out): GCAAGCTCATTAACAACAAGAC | | |
| AT1G18270-R(in): TTGGCTGGAGTTCCTTTG | | |

5' RACE Adapter sequence：GCUGAUGGCGAUGAAUGAACACUGCGUUUGCUGGCUUUGAUGAAA
